# Explore the Functional Connectivity between Brain Regions during a Chemistry Working Memory Task

**DOI:** 10.1371/journal.pone.0129019

**Published:** 2015-06-03

**Authors:** Wen-Chi Chou, Jeng-Ren Duann, Hsiao-Ching She, Li-Yu Huang, Tzyy-Ping Jung

**Affiliations:** 1 Institute of Education, National Chiao-Tung University, Hsinchu, Taiwan; 2 Biomedical Engineering Research Center and Graduate Institute of Clinical and Medical Science, China Medical University, Taichung, Taiwan; 3 Swartz Center for Computational Neuroscience, Institute for Neural Computation, University of California, San Diego, San Diego, CA, United States of America; Wadsworth Center, UNITED STATES

## Abstract

Previous studies have rarely examined how temporal dynamic patterns, event-related coherence, and phase-locking are related to each other. This study assessed reaction-time-sorted spectral perturbation and event-related spectral perturbation in order to examine the temporal dynamic patterns in the frontal midline (F), central parietal (CP), and occipital (O) regions during a chemistry working memory task at theta, alpha, and beta frequencies. Furthermore, the functional connectivity between F-CP, CP-O, and F-O were assessed by component event-related coherence (ERCoh) and component phase-locking (PL) at different frequency bands. In addition, this study examined whether the temporal dynamic patterns are consistent with the functional connectivity patterns across different frequencies and time courses. Component ERCoh/PL measured the interactions between different independent components decomposed from the scalp EEG, mixtures of time courses of activities arising from different brain, and artifactual sources. The results indicate that the O and CP regions’ temporal dynamic patterns are similar to each other. Furthermore, pronounced component ERCoh/PL patterns were found to exist between the O and CP regions across each stimulus and probe presentation, in both theta and alpha frequencies. The consistent theta component ERCoh/PL between the F and O regions was found at the first stimulus and after probe presentation. These findings demonstrate that temporal dynamic patterns at different regions are in accordance with the functional connectivity patterns. Such coordinated and robust EEG temporal dynamics and component ERCoh/PL patterns suggest that these brain regions’ neurons work together both to induce similar event-related spectral perturbation and to synchronize or desynchronize simultaneously in order to swiftly accomplish a particular goal. The possible mechanisms for such distinct component phase-locking and coherence patterns were also further discussed.

## Introduction

A brain connectivity study may involve anatomical, functional, and effective connectivity aspects [1–4]. Anatomical connectivity refers to how the physical or structural (synaptic) networks associate with other physiological structures and connect with each other via physical links between sets of neurons. It focuses on the relationships and connections between individual neurons [5]. Functional connectivity refers to the connections that reflect temporal coherence between spatially distinct neurons [2]. The main distinction between anatomical and functional connectivity is time-dependence. Functional connectivity calculates the temporal coherence between brain regions regardless of whether these regions are connected with each other via direct structural linking. Correlation, spectral coherence, and phase-locking can all be utilized to assess functional connectivity [4, 5]. Effective connectivity refers to the causal effect between neuron systems [2, 4]. As is true for functional connectivity, effective connectivity is also time-dependent. However, effective connectivity requires specific causal models to demonstrate that the structural elements can estimate causal interactions between brain regions [4].

Several approaches, including magnitude squared coherence (MSC), event-related coherence (ERCoh), and phase-locking, were developed to measure functional connectivity [6–8]. For example, MSC measures the linear covariance between two spectra at a specific frequency [6]. MSC changes are thus derived from both amplitude and phase; furthermore, MSC can be affected by either power or phase changes [9]. As a result, MSC does not separate the effects of power and phase between the interrelations of two signals. ERCoh was used to estimate phase synchronization between component activities and normalized this function by reducing the power influence through normalized the power of a given frequency at each trial [10]. ERCoh reveals the relationships between different brain regions at specific frequencies across time courses. More specifically, it focuses on those relationships that are related to the specific event’s cognitive function [11–13]. Phase-locking allows measurement of the phase synchrony of given signals [8]. Such measurements are taken from specific frequencies across time courses. Examining the phases in neural oscillations can provide a more precise understanding of the connection dynamics as well as what actually occurs when neurons fire together. Phase-locking therefore serves as an indicator of the dynamic phase interactions and relationships between brain regions independently of the effect of their power [14]. Thus, this study used ERCoh and phase-locking to explore the extent of the functional connectivity between different brain regions.

A previous study has reported enhanced theta phase-locking or theta synchronization between the frontal and posterior channels during the working memory retention stage [15]. Fell et al. examined the theta coherence between the hippocampus and the rhinal cortex of medial temporal lobe while subjects performed a word list memorized during the working memory task [16]. Their results showed enhanced theta coherence in the rhinal-hippocampal during a successful working memory process encoding stage. Anderson et al. investigated the theta coherence between the medial temporal lobe (MTL) and the prefrontal cortex (PFC) during a verbal free recall working memory task [17]. Their results showed that theta coherence increased between the MTL and PFC during the free recall stage and further reported that, according to the granger causality analysis, the increased coherence was mainly driven from the MTL to PFC. Brookes et al. studied the Sternberg working memory task with MEG and reported that frontal theta power increased and posterior theta power decreased as memory load increased [18]. In addition, Raghavachari et al. investigated the theta synchronization by intracranial EEG (iEEG) while subjects were performing the Sternberg working memory task [19]. They reported significant theta synchronization within the occipital cortex, the parietal lobe, the temporal lobe, and the frontal cortex. In other words, theta synchronization was observed at neighboring electrode locations less than 40 mm apart; furthermore, there was no significant theta synchronization when the distance of the electrodes was more than 40 mm. Thus, Raghavachari et al. concluded that theta synchronization tended to decay with distance during the Sternberg working memory task [19]. Klimesch et al. observed increasing alpha phase-locking within the posterior channels during the recognition period in good memory performers [20]. Babiloni et al. reported that beta and gamma functional connectivity between frontal and parietal were observed during the retention stage of the working memory task [21]. Though these studies reported different frequency band phase-locking, coherence, and synchronization patterns at different areas, there is still a lack of studies that have systematically examined the functional connectivity across brain regions across different frequency bands during a working memory task, particularly for the Sternberg working memory task.

These ERCoh and phase-locking studies reported above assessed the functional connectivity from the channel aspect. The ERCoh and phase-locking of channel data have been known to be biased by volume conduction in EEG recordings [22]. Delorme and Makeig [10] suggested that ICA is effective to decompose the brain and non-brain source that was summed by volume conduction in scalp EEG and, furthermore, might be helpful in identifying different brain generators of the EEG. ICA decomposes multi-channel EEG recordings into independent components that arise from different brain sources [23]. It seems more reasonable to test the functional connection between different sources as opposed to testing the functional connection between mixtures of sources. In addition, we would like to point out that the papers by Makeig et al. [22], Delorme and Makeig [10], Onton et al. [24], and Hipp et al. [25], they have all demonstrated that it is possible to deal with the problem of volume conduction by minimizing the influence of other independent sources to the target component processes using independent component analysis (ICA). On the other hand, Hipp et al., [25] also proposed that ICA could be powerful to reject artifactual components. These studies provided strong evidence that ICA preprocess can be used to decompose the EEG or MEG data by spatially transforming the raw recordings into source components with minimal mutual information among them. Therefore, the ICA preprocess exhibited an appropriate preprocessing step before computing the functional connectivity between different brain processes. This study thus took the advantage of the preprocessing step. In addition, there is a lack of studies which examine component ERCoh and component phase-locking simultaneously during a working memory task (more specifically, during an encoding process across different frequency bands, different time courses, and different brain regions). The component event-related coherence (ERCoh) and phase-locking were used in this study to explore the interactions between the independent EEG components in order to reveal their functional connectivity between different brain regions. Therefore, combining both component ERCoh and component phase-locking might allow a more accurate reflection of genuine functional connectivity between different brain regions. One of the aims of this study is to investigate ERCoh and phase-locking based on the independent components conducted during an Independent Component Analysis (ICA). In doing so, we expect to develop a better understanding of the functional connectivity between different brain EEG processes with fewer contaminations from irrelevant artifact and volume conduction.

With respect to the temporal dynamic patterns, there are many studies which have examined the various frequency patterns during a working memory task [26–30]. Meltzer et al. reported that the theta and alpha increases in the frontal midline cortex while the alpha decreases in the occipital cortex during a working memory operation [30]. In addition, some studies have reported that frontal theta activities are involved in the memory encoding process and, furthermore, that parietal and occipital theta functions were associated with working memory tasks [19, 31, 32]. These studies reported very interesting temporal dynamic patterns during working memory tasks. However, few studies have investigated the functional connectivity between different brain regions or the extent to which the functional connectivity pattern was related to the temporal dynamic activity between different brain regions.

The previous studies have reported that the primary visual sensory cortex located in the occipital region has reciprocal inter-cortical connections with the anterior cortical areas of the system of central executive function. These connections are responsible for involvement in the selection of actions and for maintaining working memory in order to process sensory information in detail [33–35]. Other studies have reported that the parietal lobe is responsible for the capacity limit of short-term memory [36], or memory load [37]. This study purposely sought, then, to examine whether and how the occipital region interact with the frontal midline, and how the occipital region interact with the central parietal region to accomplish the encoding of complicated chemistry concepts. In addition, this study also examined the reaction-time-sorted spectral perturbation (RSSP) and event-related spectral perturbation (ERSP) over selected brain regions (frontal midline, central parietal and occipital cluster). Moreover, the other aim of this study is to examine whether and how the component temporal dynamics are in line with the functional connectivity patterns. This approach will broaden our understanding of how different brain regions coordinate in order to accomplish chemistry-related working memory tasks. By studying the interrelationships between these temporal dynamics and functional connectivity, we expect to advance our understanding of how brain regions collaborate during a chemistry working memory task. In order to reach our goals, the following research questions serves to guide this study: (1) How do the temporal dynamic patterns in the frontal midline (F), central parietal (CP), and occipital (O) regions differ from each other across theta, alpha, and beta frequencies during a chemistry working memory task? (2) Does any component coherence/phase-locking exist between F-CP, CP-O, and F-O across different frequency bands? (3) Are the temporal dynamic patterns consistent with the functional connectivity patterns across different frequencies and time courses?

## Materials and Methods

### Subjects

Sixty-four undergraduate science majors were recruited for this study. There were 36 males and 28 females whose age ranged from 18 to 22 years old (mean 19.1 years). All subjects are right-handed. All students were recruited through advertisements on the school bulletin board. This study was approved by the Institutional Review Board of China Medical University Hospital, and all participants were paid for their participation. Before the start of the experimental procedure, all participants were asked to read and sign a consent form.

### Instrument

EEG data were recorded using NeuroScan SynAmps2 (Compumedics, Melbourne Australia), and the stimuli were delivered through STIM2. The EEG data were recoded from 62 scalp electrodes and 4 bipolar electrooculography (EOG) electrodes. The sampling rate of EEG data collection was 1,000 Hz with analog band-pass from DC to 100 Hz; the electrode impedance was kept below 5 kΩ during the data recoding period. All electrodes were referenced to linked mastoids (M1 and M2) and followed the extended international 10–20 system locations.

### Experimental design and procedure

This study was specifically designed to explore how students encode, maintain, and retrieve chemistry concepts in a Sternberg paradigm design [38]. It allowed us to study working memory processes during science learning, particularly how students encode concrete chemistry concepts in text mode presentations.

**Fig 1:**
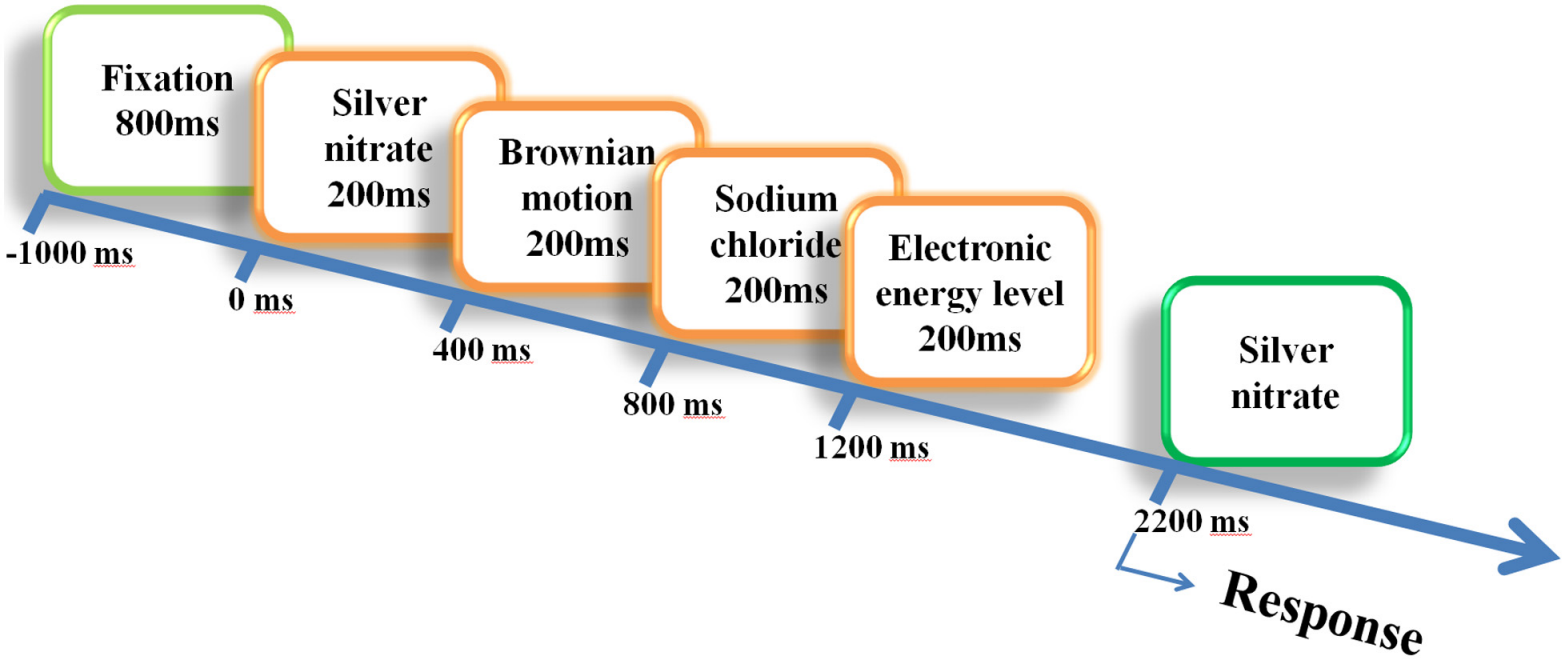
Procedure timeline. The experimental procedure in this study followed the Sternberg task paradigm. Each trial consisted of four stimuli and one target probe. Subjects were asked to determine whether the probe had appeared in the previous four stimuli.

In this study, a total of 60 chemistry concepts were developed as experimental stimuli in word presentation. The participants had already previously learned all of the concepts that were selected from their middle and high school chemistry textbooks. For each trial, 4 out of the 60 concepts were chosen as stimuli and presented sequentially for 200 ms each; the 60 scientific concepts each appeared once as the probe. After all stimuli were presented, a 600ms maintenance period accompanied with a blank screen was displayed. The probe then was presented for 200ms, and the subjects were asked to respond as to whether or not the probe had appeared in the previous four stimuli (Fig 1). Subjects were asked to respond either “Yes” by pressing the left mouse button or”No” by pressing the right mouse button. Reaction time (RT) was defined as the time interval between the probe onset and the moment the subject pressed a button.

### Data analysis

#### Independent component analysis (ICA) and dipole source localization

The open source EEGLAB toolbox running under the Matlab 7.11 platform was used to analyze the EEG data (http://sccn.ucsd.edu/eeglab/). The EEG data was digitally filtered to retain frequencies between 0.5 Hz and 52 Hz and then down-sampled to 250 Hz High frequency muscle noise and irregular artifacts were removed through visual inspection. All remaining data were analyzed with an extended infomax Independent Component Analysis (ICA) [23, 39, 40–42] by using the “runica” function [43] under the EEGLAB toolbox.

ICA is a useful method for decomposing brain and non-brain activity from a mixture of EEG recordings [10, 22,23, 39]. The EEG signals were recorded through the electrodes on the scalp and included background noise, irregular artificial activity, and brain activity that were generated from multiple brain sources. ICA assumes that the temporal brain activities from multiple brain sources are statistically independent, and that in order to find an unmixing weight matrix (W) to decompose the EEG channel data (x) into the statistically independent components activation (u): u = Wx. The output number of components is equal to the input number of channels, and the output time points of components activation (time points of u) are equal to the time points of input EEG channel recordings (time points of x) [23, 39]. A mixture of brain activities collected from the EEG scalp channels could be decomposed into independent components arising from different brain and artifactual sources. The dipole fitting process was performed in order to localize equivalent dipole sources, represented by three-dimensional coordinates (x, y, z), for each component based on the component’s scalp topography. The process is based on the boundary element head model [44] and implemented in the DIPFIT2 routine as a plug-in to the EEGLAB toolbox. The components with equivalent dipole locations inside the head sphere and with residual variance below 15% were selected for further analysis. After diploe source localization, the EEG data were separated into non-overlapping epochs of 5.7 sec. Each epoch was segmented from 1 sec before the first stimuli onset to 4.7 sec after the first stimuli onset (-1000~4700 ms). The time interval from -1000 to 0 ms was used as a baseline. Each epoch consisted of four stimuli, one probe, and a response period. Only the epochs that generated correct responses were selected for further analysis. The maximum and minimum numbers of correct epochs were 58 and 25 (on one subject), and the average number of correct epochs across subjects was 47.2.

#### Independent component clustering analysis

The independent component (IC) clustering and group analysis was conducted with the STUDY function from the EEGLAB toolbox [10, 45]. The clustering analysis includes the following steps. First, the component spectrum power, event-related potential (ERP), event-related spectral perturbation analysis (ERSP) [46], inter-trial coherence, and scalp topography of each independent component were computed. Second, with the exception of the dipole source location that was featured in only 3 dimensions (x, y, z), other measures were compressed into a 10-dimension vector using principal component analysis (PCA) [10, 45], resulting in a 53-dimensional feature space. Finally, the *K*-mean clustering in the EEGLAB was used to group the independent components from all subjects into 40 clusters. In order to normalize the variance from the different measures in the 53-dimension feature vectors, the dipole location was given a weight of 20, ERSP was given a weight of 9, and the other measures (spectrum, ERP, inter-trial coherence, and scalp topography) were each given a weight of 1. ICs whose distance to the cluster centroid was greater than three standard deviations away from the cluster centroid were removed; those clusters, which mainly account for noise, eye movement, or muscle signal components, would be discarded. Finally, approximately 10–13 distinct clusters resulted from the initial 40 clusters. The component clustering process is used to identify the components with highly comparable power spectra activity patterns, comparable scalp projections, and dipole source locations from multiple subjects. In other words, it is used to group a set of comparable components across multiple subjects into a cluster [24, 39, 45]. In addition, Onton et al. also demonstrated that clusters that represent reproducible EEG activities across subjects might allow us to investigate the nature of subject differences within and between clusters [24]. This study aims to explore the connectivity between different independent brain processes that are involved in working memory processes. Therefore, identified clusters of the frontal midline, central parietal, and occipital were selected and further analyzed.

#### Reaction-time sorted spectral perturbation (RSSP) and event-related spectral perturbation (ERSP)

After clustering, a reaction-time sorted spectral perturbation (RSSP) plot was used to reveal the spectral changes of the selected clusters (frontal midline, central parietal, and occipital cluster) at a certain frequency (theta: 5 Hz; alpha: 10 Hz; beta: 15 Hz). An RSSP plot is a 2-D transformed spectral representation in which the power spectra at a certain frequency of data epochs were first sorted according to reaction time, then color-coded and stacked, and finally smoothed across adjacent trials to form a 2-D image [39]. In addition, event-related spectral perturbation (ERSP) was used to visualize mean event-related spectral power changes of the selected clusters over time relative to an experimental event [46]. Calculating an ERSP requires averaging the power spectra in different frequency bands (theta: 4–7.9 Hz; alpha: 8–12.9 Hz; beta: 13–17.9 Hz). The ERSP results are then plotted as relative changes in spectral log amplitudes from the baseline in order to represent power variations [10, 46]. The RSSP and ERSP were used to visualize the variations in power spectral changes over time [39, 46], and should allow detailed exploration of the EEG temporal dynamics between the frontal midline, central parietal, and occipital cluster.

#### Component ERCoh (Component Event-related coherence)

Component event-related coherence (component ERCoh) is one of the methods used to assess functional connectivity by measuring the phase synchronization between components at specific frequencies. Component clustering is used to assemble the components derived from ICA decomposition that have comparable power spectra activity patterns, scalp projections, and dipole source locations from multiple subjects. Event-related coherence was proposed by Rappelsberger et al. who used it to estimate the coherence that was related to specific events across a temporal dimension [7]. This study used the EEGLAB function ERPCOH to estimate ERcoh between component activities. In our study, the time windows that were used for ERCoh consisted of 279 data points, with a sampling rate of 250. So, the time windows were approximately 1,116ms in duration, and used 3 cycles for wavelet and divided frequencies from 3Hz to 30Hz. The temporal resolution of ERCoh estimation was 8ms. The value range of ERCoh was from 0 to 1. A value of 0 indicated an absence of synchronization between the two component activities, with the extent of synchronization increasing as the value approached 1. According to Onton et al., each selected cluster might comprise different subjects’ components due to the fact that some subjects were lacking one or more independent component after ICA [24]. Accordingly, the component ERCoh was computed between the frontal midline and central parietal (F-CP) pair, the central parietal and occipital (CP-O) pair, and the frontal midline and occipital (F-O) pair with three component pairs for each subject. There were 34 subjects involved in the F-CP pair, 39 subjects in the CP-O pair, and 31 subjects in the F-O pair. The colors of the map that are red (or close to red) indicate that there was greater coherence between two component clusters (two different brain regions, for instance, F-CP) at specific frequencies.

#### Phase-locking value computation and equivalent dipole density

Phase-locking is another method which is used to measure the functional connectivity by estimating the phase synchronization between the given signals [8]. Their studies focused on channel space, whereas our study specifically focused on investigating the interactions between two independent component clusters. Phase-locking values (PLVs) were computed for three selected component pairs (F-CP, CP-O, and F-O). Lachaux et al.’s proposed procedure was adopted to compute the PLVs of the selected component pairs [8]. The time courses of the selected components for each subject were first epoched according to stimulus onset (-1~4.7 sec) and filtered using a band-pass finite impulse response (FIR) filter in a narrow band centered at specified frequencies with a bandwidth of ±2 Hz. Before filtering, the epoched EEG time courses were multiplied by a Hanning window to prevent frequency leakage. The PLVs of every single frequency bin from 4–50 Hz (47 different bins) were calculated. The narrow-band component activities were then convolved with a complex Gabor wavelet to decompose the signals into amplitude and phase components. In order to compute the PLVs, the amplitude components were not utilized for further analysis. To compute the PLVs between the components of each pair at each time point, the phase differences were calculated and summed for all of the epochs. This resulted in a PLV (t) time series for each component pair across the entire epoch length. Some of the subjects had fewer component pairs because they were lacking one or more independent component in their ICA results.

In order to test the significance of the phase-locking between component pairs at specific time periods showing significant phase-locking, a bootstrapping (with random permutation) approach was used to empirically establish the significance levels for phase-locking statistics (PLS). In our study, we adopted the method employed in Lachaux et al.’s [8] and Nichols and Holmes’s [47] papers in order to compute both the phase-locking values and the significance level of the PLV using surrogate data. We computed the distribution of the NULL hypothesis (i.e., H0: there is no PLV at all) using the surrogate data as the PLV baseline. First, the time point sequences of the epochs for each component pair were randomly shuffled. Then, we randomly shuffled the order of one EEG epoch of one component in the pair and fixed the epoch order of the other component. Finally, we computed the PLVs of the two EEG epochs using the same approach described above. This process was repeated 100 times and resulted in 100 PLV time series that were then used to compute the PLV baseline (NULL hypothesis) and its standard deviation. Taken together, the PLV baseline and standard deviation provided the significance levels of the PLV measure at each time point within an epoch. In order to compare whether the PLV that larger than significance threshold between different time points, we normalized the baseline PLV value by Z score transform, and identified the threshold PLV value through the following equation: threshold PLV value = Mean _baseline PLV value_+2.32* Std _baseline PLV value_. Here, we used a rigorous criterion—PLV larger than 2.32 standard deviations from its baseline (the probability beyond Z_2.32_ = 0.01, which is equivalent to p < 0.01)—to identify time points with significant PLVs.

In order to summarize the time periods with significant PLVs along the task performance, we segmented the entire epoch into smaller time periods at 200 ms intervals according to the experimental paradigm. After summarizing the PLV measures into small time periods, we further summarized the PLV results into the following EEG frequency bands of interest: theta (4–7 Hz), alpha (8–12 Hz), and beta (13–17 Hz). Once a time period with significant PLVs is within the specified EEG frequency band, such as 8–12 Hz for the alpha range, this time interval would be coded as showing significant phase-locking in the alpha band. Such a process may account for the possible individual differences in the EEG frequency band for different subjects. For example, some subjects may have a slightly lower alpha frequency than others. As a result, we could determine the time windows during the entire task performance that showed significant theta, alpha, or beta phase-locking.

For group analysis, we adopted the method described by Duann et al. [48]. The PLV results of the same component pairs across different individuals were pooled using a binomial test. The test was performed at each time window from the same EEG frequency band (e.g., theta, alpha, etc.) of the equivalent component pair across different subjects in order to test if the PLVs at the specific time window were consistently significant across subjects. There were 34 subjects involved in the F-CP pair, 39 subjects in the CP-O pair, and 31 subjects in the F-O pair. The average number of trials is about 47.2, while the maximum is about 58 trails and the minimum is about 25 trails. Only those time windows with PLV significance levels exceeding a threshold of p < 0.05 tested across all the subjects of each component pair were reported here.

In order to better visualize the spatial distribution of the numbers of phase-locking component pairs that existed between two brain regions that reached the significance level, the dipole density was further computed across time courses in the various theta, alpha, and beta frequencies. Dipole density was determined by first spatially warping the coordinates of the two dipoles in a component pair (e.g., F-O component pair) of a single subject onto the Montreal Neurological Institute (MNI) standard brain. Then, the transformed coordinates were spatially smoothed using a 3-D Gaussian kernel. Finally, the spatially smoothed dipole maps across subjects were summed to form the dipole density plots [49]. Seventeen component pairs in the F-O brain regions reached significance in the theta band during the stimulus 1 onset (0~200 ms). Thus, the 17 component pairs were plotted in the dipole density map. The colors of map that are red (or close to red) indicate that greater phase-locking existed between two different brain regions.

## Results

### The temporal dynamics and component ERCoh/phase-locking between central parietal and occipital regions

**Fig 2:**
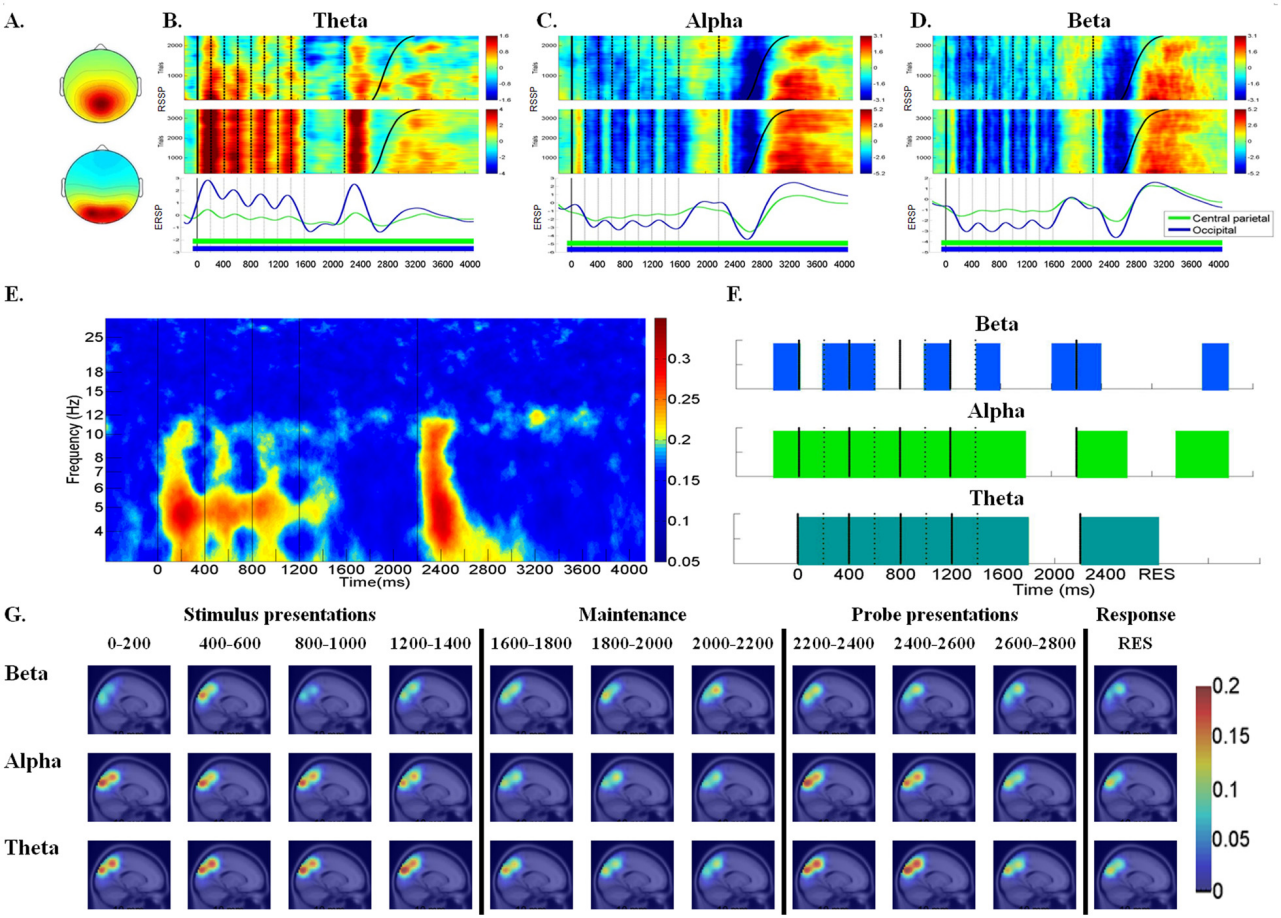
RSSP, ERSP, component ERCoh, component PLC and dipole density between central parietal and occipital regions in the theta, alpha, and beta frequencies. (A) The mean cluster scalp map of central parietal cluster (top) and occipital cluster (bottom). (B) RSSP and ERSP of the central parietal and occipital clusters in the theta frequency. (C) RSSP and ERSP of central parietal and occipital clusters in the alpha frequency. (D) RSSP and ERSP of central parietal and occipital clusters in the beta frequency. For the central parietal cluster, the RSSP in the theta band was identified as significant when powers >0.36 dB (colored in orange and red) or < -0.09 dB (colored in deep blue). In the alpha and beta band, the RSSP was identified as significant when powers > 0.69 dB or < 0.11 dB and > 0.56 dB or < 0.02 dB, respectively. For occipital cluster, the RSSP was identified as significant in the theta, alpha and beta band when powers > 0.13 dB or < -0.36 dB; > 0.40 dB or < -0.24 dB; > 0.41 dB or < -0.14 dB, respectively. In addition, the ERSP values of central parietal cluster and occipital cluster that reached statistical significance (*p* < .01) compared to the ERSP of baseline with the time intervals were marked with underline colored mark for different clusters respectively. (E) Component ERCoh between central parietal and occipital regions (F) Component PLC between central parietal and occipital regions in the theta (4–7 Hz), alpha (8–12 Hz) and beta (13–17 Hz) frequencies. The cyan, green and blue lumps indicated phase-locking that reached binomial statistical significance in theta, alpha and beta frequencies, respectively. (G) The dipole density map that presented the number of phase-locking component pairs between the central parietal and occipital regions across time courses in the theta, alpha, and beta frequencies.

Fig 2A–2D illustrates the average scalp maps, RSSP, and ERSP for theta, alpha, and beta activity in the central parietal cluster (top row) and occipital cluster (bottom row), which consisted of 53 and 76 components, respectively. The occipital cluster’s theta RSSP showed statistically significant theta power augmentation (*p* < .01) after each stimulus onset and after probe presentation (Fig 2B). Although the theta RSSP at the central parietal cluster is quite similar to the occipital cluster, the power of the central parietal cluster decreased in subsequent presentations to a much greater extent than in the occipital cluster. The ERSP patterns are consistent with RRSP, indicating that the theta frequency increased sharply after each stimulus and probe presentation (*p* < .01); however, the sharp increase in the central parietal theta frequency was more distinct during the first stimulus and after the probe onset (*p* < .01) (Fig 2B).

Regarding the alpha frequency, the occipital RSSP showed statistically significant alpha decreased (*p* < .01) after each stimulus offset and before the subjects’ responses (Fig 2C). The central parietal’s RSSP patterns were quiet similar to those observed in the occipital region; however, the alpha decreased was more pronounced during the first stimulus presentation and decreased considerably in subsequent presentations (Fig 2C). The ERSP patterns are in line with the RRSP, which indicated that there was obvious alpha decreased (*p* < .01) time locked to the stimulus offset and after probe presentation, and a salient alpha burst after the subjects’ responded (Fig 2C). For beta frequency, the overall RSSP and ERSP patterns of the central parietal and occipital region were similar to the alpha patterns. Pronounced beta power decreased (*p* < .01) was observed after stimulus and probe presentation. In addition, the beta power rebound was observed after the subjects’ responses (Fig 2D).

Fig 2E illustrates the component ERCoh between the central parietal and occipital regions (CP-O) across different frequencies. The component ERCoh showed salient theta and alpha ERCoh (*p* < .01) during the stimulus presentation and after the probe presentation (Fig 2E). The component ERCoh also showed pronounced theta ERCoh (*p* < .01) across each stimulus and probe presentation between the CP-O regions. The alpha ERCoh increased after the first stimulus presentation and decreased considerably in the subsequent stimulus presentations. The beta ERCoh, however, was not apparent. This component ERCoh was in line with the theta and alpha RSSP and ERSP patterns presented above.

Fig 2F shows the component phase-locking (component PL) comparison results between the central parietal and occipital regions (CP-O) with respect to the theta, alpha, and beta frequencies (from bottom to top) that reached statistical significance during the binomial test across time courses. The component PL results showed statistically significant theta and alpha phase-locking during each stimulus presentation and after the probe presentation, which is consistent with the component ERCoh and RSSP patterns. In order to confirm whether the PLV and ERCoh patterns were consistent with each other, the association test was performed. The results showed that component ERCoh and component PL were highly and significantly correlated (Theta: *r* = .331, *p* < .001; Alpha: *r* = .344, *p* < .001; Beta: *r* = .355, *p* < .001). The scatter beta phase-locking patterns were observed during the stimulus and probe presentations and after the subjects’ responses.

Fig 2G presents the dipole density map that showed the number of phase-locking component pairs observed between the central parietal and occipital regions (CP-O) that reached significance across time courses in the theta, alpha, and beta frequencies (from bottom to top). The dipole density map indicated that the dipole density at the theta and alpha frequencies is comparable and, furthermore, that they are both higher than the beta frequency across the stimulus presentations (0~1400 ms) and after the probe presentations (2200~2600 ms). The dipole density observed during the maintenance period (1800~2200 ms) was lower than that observed during the stimuli and probe presentations across different frequencies, which is consistent with the ERCoh and PL results.

To summarize, the temporal dynamics are consistent with the component ERCoh, the component PL, and the dipole density patterns. Our results contribute interesting evidence of the salient functional connectivity that exists between the central parietal and occipital regions during the stimulus and probe presentation of a working memory task in both the theta and alpha frequency band.

### The temporal dynamics and component ERCoh/phase-locking between frontal midline and occipital regions

**Fig 3:**
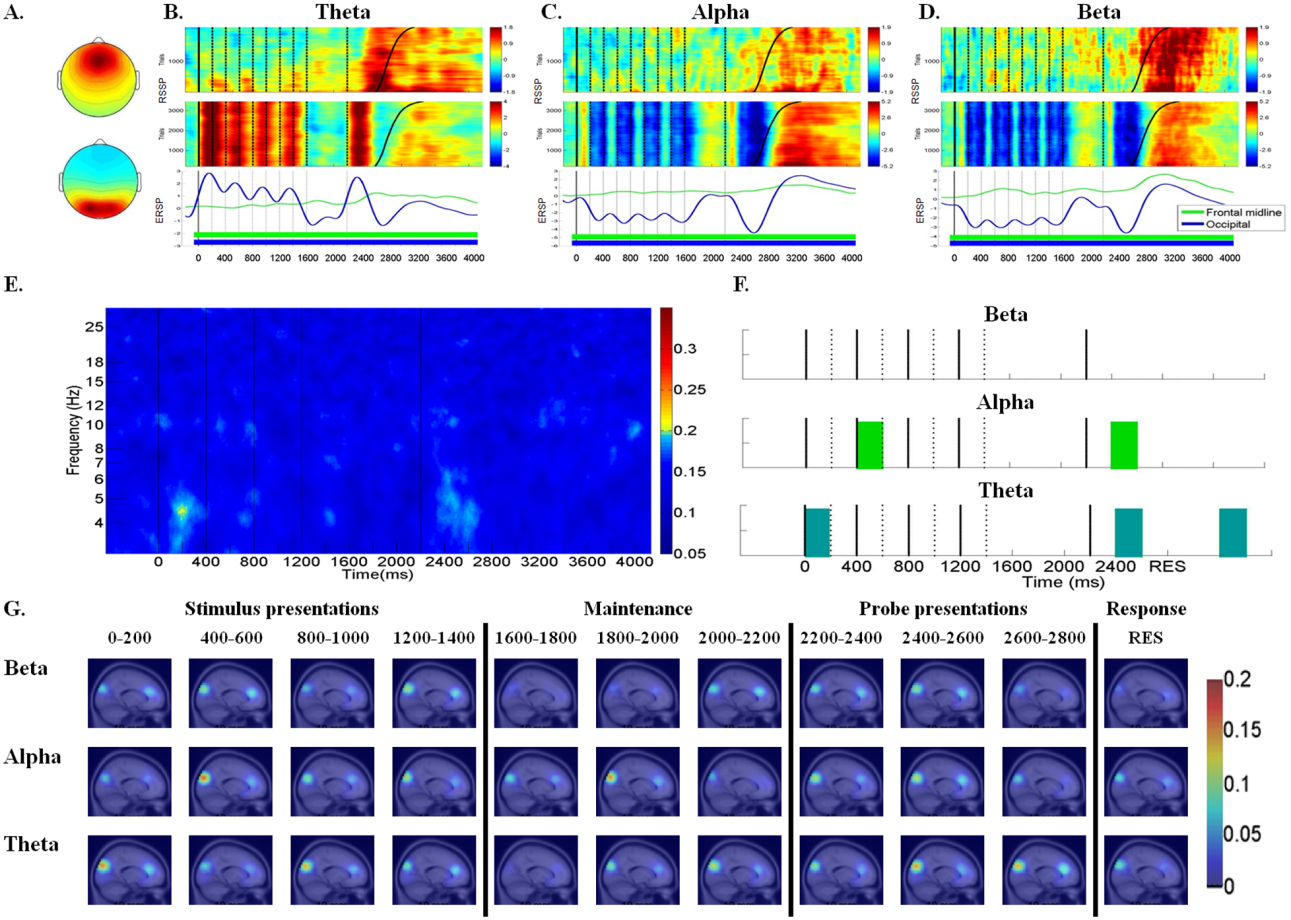
RSSP, ERSP, component ERCoh, component PLC and dipole density between frontal midline and occipital regions in the theta, alpha, and beta frequencies. (A) The mean cluster scalp map of frontal midline cluster (top) and occipital cluster (bottom). (B) RSSP and ERSP of the frontal midline and occipital clusters in the theta frequency. (C) RSSP and ERSP of frontal midline and occipital clusters in the alpha frequency. (D) RSSP and ERSP of frontal midline and occipital clusters in the beta frequency. For the frontal midline cluster, the RSSP in the theta, alpha and beta band was identified as significant when powers > 0.64 dB or < 0.06 dB; > 0.61 dB or < 0.07 dB; > 0.73 dB or < 0.12 dB, respectively. For occipital cluster, the RSSP in the theta, alpha and beta band was identified as significant when powers > 0.13 dB or < -0.36 dB; > 0.40 dB or < -0.24 dB; > 0.41 dB or < -0.14 dB. In addition, the ERSP values of frontal midline cluster and occipital cluster that reached statistical significance (*p* < .01) compared to the ERSP of baseline with the time intervals were marked with underline colored mark for different clusters respectively. (E) Component ERCoh between frontal midline and occipital regions (F) Component PLC between frontal midline and occipital regions in the theta (4–7 Hz), alpha (8–12 Hz) and beta (13–17 Hz) frequencies. The cyan, green and blue lumps indicated phase-locking that reached binomial statistical significance in theta, alpha and beta frequencies, respectively. (G) The dipole density map that presented the number of phase-locking component pairs between the frontal midline and occipital regions across time courses in the theta, alpha, and beta frequencies.

Fig 3A–3D illustrates the average scalp maps as well as the RSSP and ERSP for theta, alpha, and beta activity in the frontal midline cluster (top row) and occipital cluster (bottom row), which consisted of 44 and 76 components, respectively.

The RSSP and ERSP patterns at the frontal midline cluster were very different than those observed at the occipital cluster, including theta, alpha, and beta frequencies (Fig 3B–3D). The frontal midline theta power augmentation was less pronounced (*p* < .01) during the stimuli period; furthermore, a greater degree of theta power appeared (*p* < .01) during the maintenance period (1600~2200 ms) and theta power augmentation (*p* < .01) occurred after both the probe presentation and subjects’ responses. However, the occipital cluster evidenced a regular and pronounced theta augmentation (*p* < .01) pattern following each stimuli and probe presentation. The frontal midline ERSP indicated that theta power increased (*p* < .01) slightly during the maintenance period and continued to increase until the subjects responded (Fig 3B). For the alpha and beta frequencies, an increase in alpha and beta patterns were observed (*p* < .01) in the frontal midline cluster, while decrease patterns were observed (*p* < .01) in the occipital cluster across the stimuli presentation, maintenance, and after probe presentation. Interestingly, both occipital alpha and beta decreased were observed (*p* < .01) to be time-locked to each stimuli offset and after the probe presentation. However, the occipital and frontal midline clusters all showed that the alpha bursts and the beta rebounds after the participants’ responses (Fig 3C and 3D).

The component ERCoh between the frontal midline and occipital regions (F-O) across different frequencies which showed some weak ERCoh was observed (*p* < .01) for theta frequency during the first stimulus presentation and after the probe presentation; however, the alpha and beta ERCoh was obscure (Fig 3E). The results of the component PL (Fig 3F) showed statistically significant theta phase-locking at the first stimulus onset and after the probe presentation, which is consistent with the component ERCoh patterns. The component PL results showed statistically significant alpha phase-locking at the second stimulus and after the probe presentation, which was not found in the component ERCoh results. No statistically significant phase-locking was found in the beta frequency, whether in the component ERCoh or the component PL. The association test between PLV and ERCoh was performed to examine whether the PLV pattern was consistent with ERCoh pattern, and the results showed a statistically significant correlation between ERCoh and PLV (Theta: *r* = .203, *p* < .001; Alpha: *r* = .280, *p* < .001; Beta: *r* = .305, *p* < .001). The dipole density map (Fig 3G) indicated that the dipole density at the theta frequency showed higher dipole density during the first stimulus presentation and after the probe presentation (2400~2800 ms) than during other time periods, which is consistent with the component ERCoh and the component PL. The higher dipole density at the alpha frequency was found during the second stimulus and probe presentations and response, which is consistent with the component PL component results.

To summarize, the temporal dynamics showed very different patterns between the frontal midline and occipital regions, which is generally in line with the results of the component ERCoh and the component PL as well as the dipole density results that suggested less functional connectivity exists between these two regions. There was, however, one exception: the component ERCoh and component PL for the theta frequency all consistently demonstrated that functional connectivity existed during the first stimulus and after the probe presentation.

### The temporal dynamics and component ERCoh/phase-locking between the frontal midline and central parietal regions

**Fig 4:**
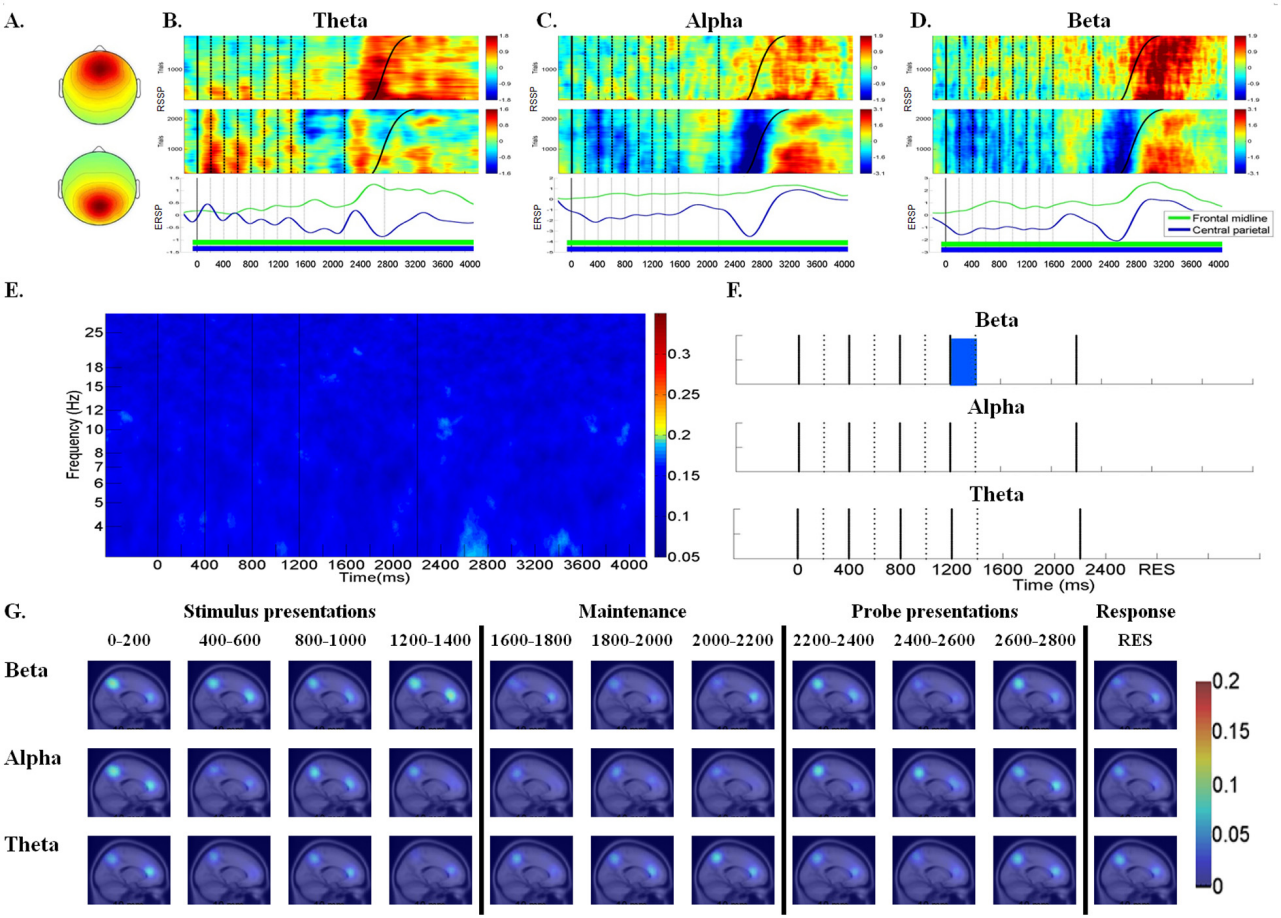
RSSP, ERSP, component ERCoh, component PLC and dipole density between frontal midline and central parietal regions in the theta, alpha, and beta frequencies. (A) The mean cluster scalp map of frontal midline cluster (top) and central parietal cluster (bottom). (B) RSSP and ERSP of the frontal midline and central parietal clusters in the theta frequency. (C) RSSP and ERSP of frontal midline and central parietal clusters in the alpha frequency. (D) RSSP and ERSP of frontal midline and central parietal clusters in the beta frequency. For the frontal midline cluster, the RSSP in the theta, alpha and beta band was identified as significant when powers > 0.64 dB or < 0.06 dB; > 0.61 dB or < 0.07 dB; > 0.73 dB or < 0.12 dB, respectively. For central parietal cluster, the RSSP in the theta, alpha and beta band was identified as significant when powers > 0.36 dB or < -0.09 dB; > 0.69 dB or < 0.11 dB; > 0.56 dB or < 0.02 dB, respectively. In addition, the ERSP values of frontal midline cluster and central parietal cluster that reached statistical significance compared to the ERSP of baseline with the time intervals were marked with underline colored mark for different clusters respectively. (E) Component ERCoh between frontal midline and central parietal regions (F) Component PLC between frontal midline and central parietal regions in the theta (4–7 Hz), alpha (8–12 Hz) and beta (13–17 Hz) frequencies. The cyan, green and blue lumps indicated phase-locking that reached binomial statistical significance in theta, alpha and beta frequencies, respectively. (G) The dipole density map that presented the number of phase-locking component pairs between the frontal midline and central parietal regions across time courses in the theta, alpha, and beta frequencies.

Fig 4A and 4D illustrates the average scalp maps and the RSSP and ERSP for the theta, alpha, and beta activity in the frontal midline cluster (top row) and central parietal cluster (bottom row), which consisted of 44 and 53 components, respectively.

The RSSP and ERSP results showed dissimilar patterns between the frontal midline cluster and the central parietal cluster, regardless of theta, alpha, or beta frequency (Fig 4B–4D). The frontal midline cluster’s RSSP exhibited greater theta power augmentation (*p* < .01) during the maintenance period and greater theta power augmentation after both the probe presentation and subjects’ responses. The frontal midline cluster’s ERSP clearly demonstrated that theta power increased during the maintenance period (*p* < .01) and continued to increase until the subjects responded (Fig 4B). The central parietal cluster’s RSSP and ERSP patterns showed more pronounced theta power augmentation (*p* < .01) at the first stimulus and decreased considerably both in subsequent presentations and after probe presentations (Fig 4B). The frontal midline cluster’s alpha and beta RSSP and ERSP indicated that alpha and beta power slightly increased during the stimuli presentation, maintenance, and after the probe presentation, while the central parietal cluster exhibited a decrease in power. However, the central parietal cluster’s RSSP and ERSP results showed the most pronounced alpha and beta decreased (p < .01) at the first stimulus presentation and after the probe presentation (Fig 4C and 4D).

The component ERCoh between the frontal midline and central parietal regions (F-CP) showed blurred patterns across time courses, regardless of theta, alpha, and beta frequency (Fig 4E). Similarly, there is no statistically significant component PL between the frontal midline and central parietal (F-CP) regions, whether in the theta or alpha frequency. The only statistically significant phase-locking appeared in the beta frequency during the fourth stimulus onset (Fig 4F). The association test between PLV and ERCoh was performed to confirm whether the PLV and ERCoh patterns were consistent with each other. The results showed a statistically significant correlation between ERCoh and PLV (Theta: *r* = .214, *p* < .001; Alpha: *r* = .277, *p* < .001; Beta: *r* = .256, *p* < .001). The dipole density map at the theta, alpha, and beta frequencies was consistent with the component ERCoh and component PL patterns. Only the dipole density of the beta frequency was more distinct during the fourth stimulus onset (Fig 4G).

In summary, the RSSP and ERSP results indicated that the temporal dynamics in the frontal midline cluster were very different from those in the central parietal cluster. In general, the component ERCoh, component PL, and dipole density patterns for F-CP consistently did not show obvious functional connectivity across time courses, in both the theta and alpha frequencies.

## Discussion

Previous studies either focused on the associated EEG power oscillations or ERCoh/phase-locking alone; however, few studies combined both types of data. The earlier studies tended to employ either channel ERCoh or phase-locking approaches to study functional connectivity, but very few studies ever combined both approaches together to advance the studies of functional connectivity with respect to component ERCoh and component phase-locking. This study tries to broaden our understanding of how temporal dynamics are associated with phase-locking/ERCoh by examining component ERCoh and component phase-locking together in order to better understand the functional connectivity between brain regions.

### Functional connectivity between central parietal and occipital regions

Our temporal dynamics results revealed that theta power arises instantly at stimulus and probe onset and drops sharply afterwards across all stimulus and probe presentations at both the central parietal and occipital regions. This pattern has been reported by Raghavachari et al. as theta gating phenomena [19, 27]. They suggest that gated theta is more likely to organize multi-item working memory to precisely control the cognitive demands of the working memory task. Our theta component ERCoh and component PL results further demonstrated an obvious theta functional connectivity observed in the adjacent posterior brain regions (between the central parietal and occipital regions) during the stimulus and probe presentation periods. Raghavachari’s iEEG study reported the similar patterns that theta coherence was widely distributed throughout the brain regions and higher theta coherence was found in neighboring locations of the posterior regions (occipital and parietal) that were less than 40 mm away from each other [19].

In addition, a precise theta (4–7.9 Hz) augmentation time locked to the stimulus and probe onset and decreased considerably in subsequent presentations was observed in both the central parietal and occipital regions. Similarly, the component ERCoh and PL were observed in a pronounced state during the stimulus and probe onset and decreased considerably in successive presentations. Fries et al. hypothesized that neuronal synchronization correlates with stimulus selection, and the firing rate was slightly lower when the stimulus was still new [50]. Their earlier study also reported that synchronization in the primary and secondary visual cortices correlates with stimulus selection [51]. Altogether, such distinct CP-O theta functional connectivity and temporal dynamics patterns suggested that central parietal and occipital neurons work simultaneously through neuron synchronization in phase in order to accomplish a highly demanding cognitive multi-item encoding task. Neurons synchronize in phase in order to allocate sufficient attention to each stimulus and probe precisely and without any delay. In addition, the theta power and component ERCoh all decrease considerably in subsequent stimulus, indicating that those neurons are efficient and parsimony. The strength of their theta power and synchronization closely correlates with the stimulus sequence to decide how much attention needs to be allocated to each of the sequential stimulus in order to maximize brain efficiency and reduce the cognitive load.

On the other hand, this study also revealed greater alpha power decreased in the posterior region and significant component ERCoh/phase-locking patterns between the central parietal and occipital regions. This result was similar to Sauseng et al.’s study that showed greater alpha ERCoh within the posterior areas during working memory tasks [52]. According to the previous studies results, power decreased and phase synchronization increases in the alpha frequency might correlate with the process of recognition [20, 53]. Klimesch et al. further proposed that the alpha frequency is associated with a certain type of encoding stage that enables access to stored information and thereby “extracts” the meaning of sensory information [54]. Several studies suggested that alpha decreased indicates the onset of an excitatory status and enables subjects to recognize stimuli, access meaningful information, access the information in LTM, and evaluate memory traces [54, 55]. Klimesch et al. further suggested that alpha decreased possibly controls the information flowing to those neural structures that represent information relevant to encoding [54]. Our findings of pronounced alpha power decreased and alpha ERCoh/phase-locking were observed for each stimulus and probe presentation. It is possible that the reason neurons at CP-O synchronize in phase is to direct the information flow to the task-relevant brain regions in order to encode such multi-items tasks and retrieve the crucial momentarily stored sensory item successfully.

In addition, our results showed that phase synchronization in the beta frequency was pronounced within the posterior regions (occipital and central parietal) during the stimulus and probe presentations. Tallon-Baudry et al. observed phase synchronization in the beta frequency between the visual areas during the rehearsal or maintenance of objects in visual short-term memory [56, 57]. Kopell et al. indicated that beta frequency synchronization that occurs during target processing reflects an effective integration within the attention network that involves broad brain regions [58]. Womelsdorf and Fries also mentioned that beta synchronization very likely plays a specific functional role within local visual processing areas [59]. Therefore, beta phase-locking, which was observed within the posterior regions in this study, could be attributed to the fact that the task requires visual processing involving attentional demands and mediating connectivity between task-relevant brain regions during working memory tasks.

### Functional connectivity between frontal midline and occipital region, and between frontal midline and central parietal region

The RSSP and ERSP results showed that the temporal dynamics in the frontal midline cluster were very different than those observed in the occipital cluster and central parietal cluster, regardless of theta, alpha, or beta frequency. The frontal midline theta was activated at the first stimulus and sustained the power activation during the subsequent stimulus presentations; it then slightly increased during the maintenance period and continued increasing the theta augmentation until the subjects responded. These patterns were consistent with previous studies which suggested that frontal theta activity is involved with the active maintenance of working memory information until the information is retrieved [26, 27, 31, 32] or until the execution of cognitive controls, such as rehearsal or focused attention [28, 60]. On the other hand, the occipital and central parietal regions showed pronounced theta power augmentation after stimuli and probe presentation. In summary, the frontal midline theta and occipital theta might serve two distinct functions: for the former, the function involves maintaining the process of encoding this multi-item task at the task-relevant cortical area until a response is made, while for the later, the function is to precisely allocate sufficient attention to each stimulus and probe in order to process a multi-item working memory task without any delay.

The results of component ERCoh and component PL demonstrated that theta ERCoh and phase-locking occurred at the first stimulus and after the probe presentation between the frontal midline and occipital regions. The frontal midline and occipital temporal dynamics and component ERCoh/PL pattern suggest that they are responsible for different functions; however, they do act simultaneously at a number of decisive points. The possible function of frontal midline and occipital synchronization at the critical points of the first stimulus onset and probe onset might be to enact the initiation of the encoding process and responding process by simultaneously activating these two areas’ neurons to synchronize in phase.

The alpha and beta temporal dynamic patterns were very different between the frontal and posterior regions. The component PL results showed that significant phase-locking appeared in the beta frequency during the fourth stimulus onset between the frontal and central parietal regions; furthermore, alpha phase-locking occurred at the second stimulus and after the probe presentation between F-O. Freunberger et al. suggested that long-range alpha phase synchrony was associated with the function of semantic information access in long-term memory [53]. A study reported that beta synchronization was generally related to attentional deployment [61]. Gross et al. proposed that fronto-parieto-temporal beta ERCoh is relevant for the processing of stimuli in working memory [62]. Gross et al. also proposed that visual attentional networks communicate through beta phase synchronization [62]. However, our alpha and beta phase-locking or ERCoh between F-CP and F-O is less prominent and still requires further investigation.

### Component ERCoh, component phase-locking, and dipole density

The component phase-locking that occurred within every 200 ms time window indicates that there was pronounced connectivity between the occipital and central parietal areas, and less connectivity between both the frontal and occipital areas and the frontal and central parietal areas in the theta, alpha, and beta frequencies. Similarly, the component ERCoh also revealed pronounced ERCoh patterns between the occipital and central parietal areas, and less connectivity between both the frontal and occipital areas and the frontal and central parietal areas in the theta, alpha, and beta frequencies. The dipole density maps confirm the spatial distribution where prominent phase-locking and ERCoh was consistently found across subjects.

The association test between PLV and ERCoh demonstrated that the PLV and ERCoh patterns were highly and significantly correlated with each other. This finding confirms our robust results indicating that pronounced local binding existed between the occipital and central parietal areas; furthermore, less long-range binding between the frontal and occipital areas and the frontal and central parietal areas was observed. Our results demonstrated that the measurement of phase synchronization by component phase-locking, component ERCoh, and dipole density opens a new window to advance our understanding regarding how different brain regions work collaboratively to accomplish such high-demanding multi-item working memory tasks efficiently and precisely.

## Conclusions

Previous studies have explored the functional connectivity between different brain regions by examining channel ERCoh or channel phase-locking. However, the analyses in such studies could be confounded as the EEG signals measured over the scalp electrodes are a mixture of underlying sources in the brain. The current study is a pioneer study which employed both ERCoh and phase-locking based upon the component activities together to confirm and reveal similar connectivity patterns. In addition, this paper is the first study reported that pronounced functional connectivity was observed between the occipital and central parietal regions at the theta and alpha frequencies during the scientific concepts encoding process which has not reported elsewhere. By using event-related component ERCoh and phase-locking along with the ERSP and RSSP results, we were able to investigate both the cognitive processes related to each region and also the synchronization across the different regions in the theta, alpha, and beta frequencies.

Across the theta, alpha, and beta frequencies, the occipital and central parietal showed similar temporal dynamic patterns and very pronounced component ERCoh/phase-locking during the stimulus and probe presentations. This result suggests pronounced theta temporal dynamics and phase synchronization within the posterior regions. It suggested that these neurons synchronize in phase in order to allocate sufficient attention to each stimulus and probe precisely without any delay. Furthermore, theta phase synchronization decides how much attention needs to be allocated to each of the sequential stimulus in order to maximize brain efficiency and reduce the cognitive load. In addition, salient alpha/beta power decreased accompanying pronounced functional connectivity within the posterior regions was reported. It is possible that alpha-band activities might be associated with the function of directing information to task-relevant brain regions in order to encode multi-item tasks and retrieve the crucial momentarily stored sensory item successfully. The beta-band activities might be associated with visual processing that involves attention demands as well as the mediating connectivity between task-relevant brain regions during working memory tasks.

On the other hand, the temporal dynamics and functional connectivity analysis indicated that the frontal midline region was very different from the occipital and central parietal regions in the theta, alpha, and beta frequencies. To combine the overall temporal dynamic and functional connectivity patterns between the frontal and posterior regions, it is possible that frontal midline theta-band activities might reflect the central executive function of maintaining working memory processing and, furthermore, that occipital theta-band activities might serve the function to allocate sufficient attention to each stimulus in order to process the multi-item working memory task without any delay. The frontal midline theta-band activities and occipital theta-band activity patterns highly suggest that they serve different roles across multi-item encoding periods; however, this study demonstrated that they indeed synchronize at certain critical points, such as the first stimulus and probe presentation, in order to control and enact the initiation of the encoding process and the responding process promptly and precisely.

In sum, employing multiple approaches to investigate the functional connectivity in combination with the temporal dynamics patterns advances our understanding of how brain regions coordinate during multi-item working memory processing. This study demonstrates very robust and consistent patterns across temporal dynamics, component ERCoh, component phase-locking, and dipole density between both the occipital and central parietal regions and the frontal midline and occipital regions.
